# Myc-dependent purine biosynthesis affects nucleolar stress and therapy response in prostate cancer

**DOI:** 10.18632/oncotarget.3494

**Published:** 2015-03-30

**Authors:** Stefan J. Barfeld, Ladan Fazli, Margareta Persson, Lisette Marjavaara, Alfonso Urbanucci, Kirsi M. Kaukoniemi, Paul S. Rennie, Yvonne Ceder, Andrei Chabes, Tapio Visakorpi, Ian G. Mills

**Affiliations:** ^1^ Prostate Research Group, Centre for Molecular Medicine Norway (NCMM), Nordic EMBL Partnership, University of Oslo, Oslo, Norway; ^2^ The Vancouver Prostate Centre, University of British Columbia, Canada; ^3^ Department of Laboratory Medicine, Division of Clinical Chemistry, Lund University, Malmö, Sweden; ^4^ Department of Medical Biochemistry and Biophysics, Molecular Infection Medicine Sweden (MIMS), Nordic EMBL Partnership, University of Umeå, Umeå, Sweden; ^5^ Institute of Biosciences and Medical Technology, University of Tampere and Fimlab Laboratories, Tampere University Hospital, Tampere, Finland; ^6^ Department of Cancer Prevention, Oslo University Hospital, Oslo, Norway; ^7^ Department of Urology, Oslo University Hospital, Oslo, Norway

**Keywords:** prostate, cancer, nucleotide, transcription, metabolism

## Abstract

The androgen receptor is a key transcription factor contributing to the development of all stages of prostate cancer (PCa). In addition, other transcription factors have been associated with poor prognosis in PCa, amongst which c-Myc (MYC) is a well-established oncogene in many other cancers. We have previously reported that the AR promotes glycolysis and anabolic metabolism; many of these metabolic pathways are also MYC-regulated in other cancers. In this study, we report that in PCa cells *de novo* purine biosynthesis and the subsequent conversion to XMP is tightly regulated by MYC and independent of AR activity. We characterized two enzymes, PAICS and IMPDH2, within the pathway as PCa biomarkers in tissue samples and report increased efficacy of established anti-androgens in combination with a clinically approved IMPDH inhibitor, mycophenolic acid (MPA). Treatment with MPA led to a significant reduction in cellular guanosine triphosphate (GTP) levels accompanied by nucleolar stress and p53 stabilization. In conclusion, targeting purine biosynthesis provides an opportunity to perturb PCa metabolism and enhance tumour suppressive stress responses.

## INTRODUCTION

Prostate cancer (PCa) is the most common cancer in men in the United States and Europe [1]. Decades of research have shown that the androgen receptor (AR), a steroid hormone-activated transcription factor is a major driver of disease initiation and progression. Thus, androgen-deprivation therapy strives to perturb AR activity and complements conventional cancer therapies, such as radical prostatectomy, radio- or chemotherapy. However, progression to castration-resistant prostate cancer (CRPC) occurs frequently and is ultimately fatal [2]. Interestingly, most CRPC cases still express AR and display an active AR network [3, 4]. Amplifications or mutations in the AR gene are thought to facilitate the development of CRPC, presumably by rendering the AR sensitive to other ligands or lower concentrations of androgens [5, 6].

Naturally, the AR and its target genes have been at the centre of extensive research to explore better detection and treatment options. Recently, unbiased whole-genome approaches in PCa cell lines have defined the AR as a master regulator of cell cycle genes and core metabolic networks resulting in increased anabolic metabolism [7].

Besides the AR, genes for other transcription factors, such as c-Myc (MYC), are known to be overexpressed or amplified in PCa and to contribute to disease initiation and progression [8–10]. MYC acts as a heterodimeric transcription factor in complex with its partner protein MAX [11]. The assembled complex binds to consensus binding motifs called E-boxes and regulates the expression of its target genes [12]. Interestingly and resembling core AR functions, extensive work in various other cell line models discovered genes involved in cell cycle, ribosome biogenesis and metabolism to be among MYC's targets, highlighting its impact on proliferation and biomass accumulation [13–15].

Intriguingly, while transcriptional networks for MYC have been thoroughly and unbiasedly defined in other cell line models [16], the work performed in PCa remained scarce until lately. Recent work performed in PCa models suggests similar roles for MYC in ribosome biogenesis and amino acid metabolism [17, 18]. The contribution of MYC to disease progression, however, remains poorly understood.

In this study, we combine *in vitro* and clinical expression data to determine clinically relevant MYC-regulated transcriptional networks and assess their therapeutic potential. We show that MYC tightly regulates the expression of genes in the *de novo* purine biosynthesis pathway. Furthermore, we report for the first time that inhibition of the rate-limiting enzyme for guanine nucleotide biosynthesis, IMPDH2, promotes nucleolar stress and enhances responses to anti-androgens and androgen synthesis inhibitors.

## RESULTS

### MYC regulates the expression of a core set of genes and pathways *in vitro* and is highly correlated with their expression *in vivo*

To decipher gene networks regulated by MYC in PCa, we used a stably transfected MYC-overexpressing LNCaP cell line model recently published and further referred to as LNCaP MYC [19]. Upon stimulation with Doxycycline, MYC protein levels rapidly increased to approximately 4-fold and remained stable for at least 12 h. At the transcript level Doxycycline induced maximally an 8-fold increase at 24 hours (Figure S1A and S1B).

**Figure 1:**
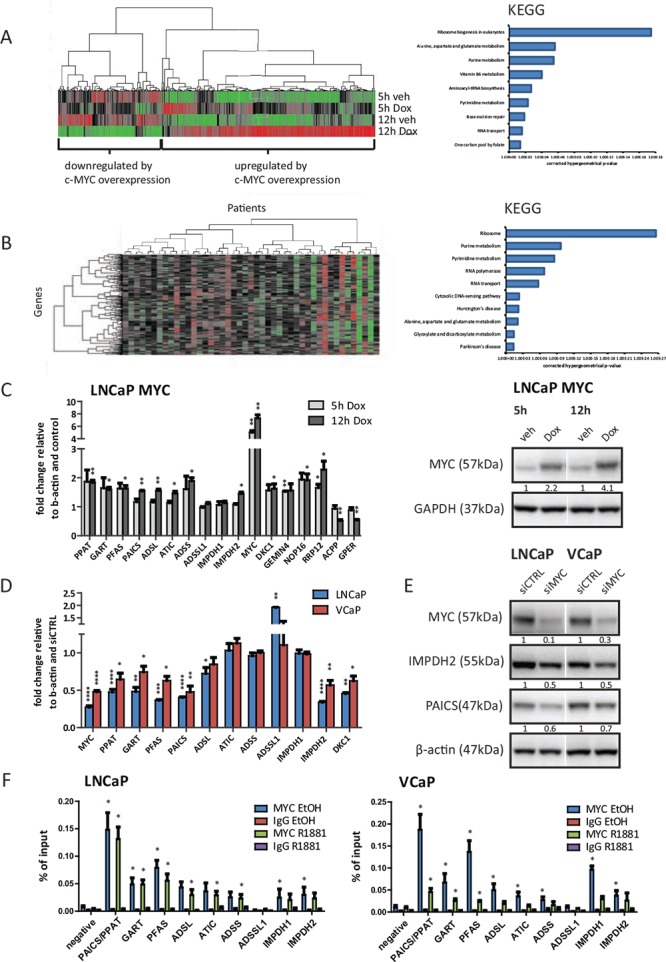
The expression of purine biosynthesis enzymes in prostate cancer cells is directly regulated by MYC **A.** Illumina beadarray results. LNCaP MYC cells were hormone-deprived for 72 h prior to stimulation with 2 μg/ml Doxycycline or vehicle for the indicated time points to induce MYC-overexpression. Total RNA of three independent experiments was isolated and subjected to expression array analysis. Genes with significantly altered expression (*q* < 0.05) compared to vehicle treated cells are displayed using unsupervised hierarchical clustering (left). Functional annotation using KEGG pathway analysis of 266 genes defined as ‘upregulated by MYC-overexpression at both time points’ is shown (right). **B.** Expression data from a previously published study (Taylor *et al*.) was used to define candidate MYC-regulated genes. Only patients marked as ‘biochemical recurrence (BCR)’ or ‘metastases (METS)’ were included in the analysis. Similarity search using Pearson correlation (1% tolerance) was performed on MYC expression and displayed using unsupervised hierarchical clustering with genes on the Y-axis and patients on the X-axis (left). Functional annotation of the 264 MYC-correlated genes using KEGG pathway analysis at 1% tolerance is shown (right) **C.** Real-time PCR results of MYC-overexpressing cells. LNCaP MYC cells were hormone-deprived for 72 h prior to stimulation with 2 μg/ml Doxycycline to induce MYC-overexpression. Total RNA was isolated, reverse transcribed and used for qRT-PCR. *n* = 3 **(D-E)** Cells were transfected with 50 nM of control or MYC siRNA for 72 h. **D.** Real-time PCR results of MYC-depleted cells. Total RNA was isolated, reverse transcribed and used for qRT-PCR. *n* = 3–4 **E.** Western Blot results of MYC-depleted cells. Protein lysates were harvested, separated by SDS-PAGE and blotted for the indicated proteins. Protein levels were normalized to siCTRL and β-actin levels. Densitometry analysis of three biological replicates with SEM can be found in Figure S1H. **F.** ChIP-qPCR detection of promoter regions of purine biosynthesis genes in MYC ChIP samples. Cells were hormone-deprived for 72 h prior to stimulation with 1 nM R1881 or vehicle for 4 h. Subsequently, chromatin was crosslinked, sonicated and subjected to ChIP using an antibody against MYC or an unspecific IgG control. *n* = 3.

Androgen treatment of LNCaP reduced MYC expression levels as previously reported (Figure S1F and S1G) [20]. Consequently, to determine the MYC-regulated transcriptome, we cultured LNCaP MYC cells in the absence of androgens prior to stimulation with Doxycycline for 5 h and 12 h. These conditions were chosen to capture primarily direct MYC-mediated effects on transcription whilst limiting AR activation. Using an unbiased Gene Set Enrichment Analysis (GSEA) approach, we found SCHUHMACHER_MYC_TARGETS_UP to be the top upregulated gene set at 12 h amongst the entire c2: curated gene sets (Figure S1C and Table S3 for Top 10 up- and downregulated gene sets). Kyoto Encyclopedia of Genes and Genomes (KEGG) analysis using a web-based tool [21] (http://genecodis.cnb.csic.es/) on the 266 genes significantly upregulated by MYC overexpression at both time points (Table S1) revealed pathway enrichment for ribosome biogenesis, amino acid metabolism and nucleotide metabolism (Figure 1A).

In parallel, to identify MYC co-expressed genes in clinical samples we downloaded clinical expression data and applied a Pearson correlation coefficient with a 1% tolerance seeded on MYC [22]. The dataset consisted of metastatic PCa cases and primary tumours for which there was evidence of biochemical relapse (BCR). This approach identified a total of 264 genes that were tightly linked to MYC expression levels in those patients (Figure 1B and Table S2). We then applied this gene set to Genecodis and observed a strikingly similar pathway enrichment to the one derived from MYC-dependent genes in our cell line dataset (Figure 1B), indicating that MYC-dependent genes are highly conserved between tissue and cell lines.

Next to ribosome biogenesis, for which a role of MYC has previously been reported [17], purine metabolism and especially the *de novo* purine biosynthesis was the most significantly enriched pathway both *in vitro* and *in vivo* (Figure 1A/B). The most consistently overexpressed genes within this pathway across 16 clinical expression array datasets were PAICS and IMPDH2, as determined by the Oncomine microarray database (Figure S1D). PAICS correlates with MYC expression in the Taylor dataset with an R^2^ of 0.625 and IMPDH2 with an R^2^ of 0.680 (Figure S1E). These are even tighter correlations than the one observed for the AR and its well-established target gene KLK3 (*R*^2^ = 0.352) and similar to the previously reported correlation between ERG and its strong target gene TDRD1 (*R*^2^ = 0.734) (Figure S1E) [24].

### The expression of genes involved in purine metabolism in prostate cancer cells is controlled by MYC

Subsequently, we moved on to PCa cell lines and initially explored whether enzymes in this pathway were androgen-dependent. We treated hormone-deprived LNCaP and VCaP cells with the synthetic androgen R1881 (1 nM), which activates the AR. Strikingly, no significant increase in transcript levels of any of the genes in the pathway could be seen after 5 h or 12 h treatment (Figure S1F). We also blotted for two genes in the pathway, PAICS and IMPDH2, and observed no significant changes on a time course from 6–72 hours (Figure S1G). By contrast, KLK3, an established AR target gene, was significantly induced by the treatment, both at the protein and mRNA levels (Figure S1F and S1G).

Next we assessed the role of MYC in the transcriptional regulation of these enzymes. An obligate dependency on MYC would imply that expression levels of target genes would mirror changes in MYC levels, both upon overexpression and knockdown. First, we used the abovementioned LNCaP MYC overexpressing model to validate our microarray predictions and assessed transcript changes after 5 h and 12 h of MYC overexpression. As positive controls, we selected two highly ranked genes, NOP16 and DKC1, which are also previously reported MYC target genes [25, 26], and two top up- and downregulated genes from our microarrays (RRP12 and GEMIN4 and ACPP and GPER, respectively). Strikingly, with the exception of *ADSSL1* and *IMPDH1,* all *de novo* purine biosynthesis and IMP-converting enzymes showed a significant (*P* < 0.05) increase in their mRNA levels upon MYC overexpression of a similar magnitude to the positive controls (Figure 1C). At the protein level, no significant changes could be observed at the 5 h or 12 h timepoints (data not shown). However, the appropriate timepoints to assess protein changes relative to transcript changes are challenging to predict since other factors, such as the availability of metabolites or the conformation and stability of the enzymes in response to substrate binding, can affect the protein levels of these enzymes, too.

MYC target genes have previously been reported to be responsive to MYC knockdown [17]. Consequently, we used siRNA against MYC and achieved a knockdown of about 70% in LNCaP cells and of 50% in VCaP cells at the mRNA level and about 70% at the protein level in both lines (Figure 1D/E and S1H). This led to a significant (*P* < 0.05) reduction of the mRNA levels of PPAT, GART, PFAS, PAICS, ADSL and IMPDH2 in both cell lines with the established MYC-target DKC1 serving as a positive control (Figure 1D).

Using Western blotting, we also observed a strong and reproducible reduction (30–40%) in the protein levels of PAICS and IMPDH2 (Figure 1E and S1H).

The tight co-expression of these enzymes with changes in *MYC* expression implies that these genes may be directly regulated by MYC. It has recently been reported that MYC binds preferentially to the proximal promoters of target genes and a number of MYC ChIP-seq datasets are available through the ENCODE initiative for cell lines, although strikingly not PCa cell lines [27]. We identified overlapping MYC consensus binding sites in a minimum of three cancer cell lines lying at the transcription start sites (TSS) of every gene in the pathway (Figure S2). To determine whether these were indeed sites of MYC recruitment in PCa cell lines (LNCaP and VCaP), we designed primers against these consensus sites (Figure S2A and S2B).

Subsequently, we performed chromatin immunoprecipitation (ChIP) in the presence and absence of androgens using an antibody against MYC. Strikingly, MYC bound significantly (*p* < 0.05) to the TSS of all genes in the purine biosynthesis pathway with the exception of *ADSSL1*, thereby suggesting a direct and global regulation of purine biosynthesis in PCa cell lines (Figure 1F). In conditions of androgen treatment, MYC levels were significantly reduced in the VCaP cell line and MYC enrichment at the TSS was concomitantly lower.

**Figure 2:**
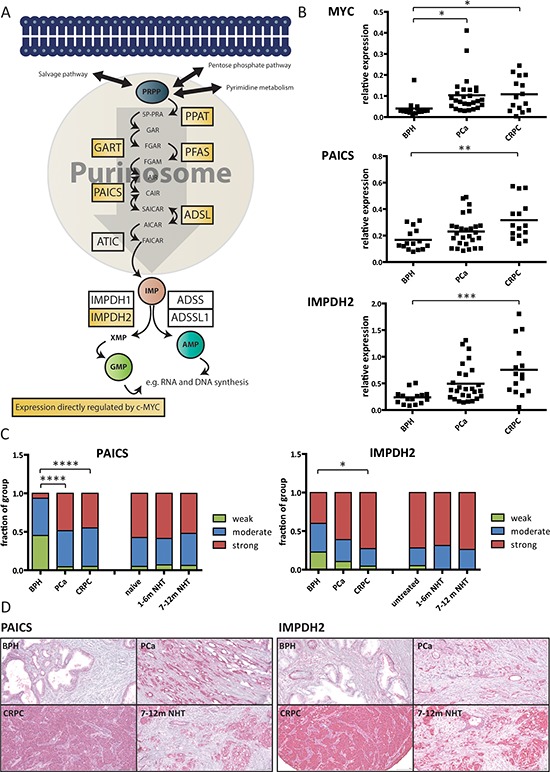
PAICS and IMPDH2 are overexpressed in prostate cancer patients **A.** Schematic overview of the *de novo* purine biosynthesis pathway with direct MYC targets highlighted in yellow. **B.** Real-time PCR results on clinical samples. Total RNA was collected from prostate biopsies of patients suffering from benign hyperplasia (BPH), prostate cancer (PCa) or castrate-resistant prostate cancer (CRPC). The expression levels of MYC, PAICS and IMPDH2 were measured using real-time PCR and normalized to the expression of TATA-box binding protein (TBP). The line displays the mean value and significance was determined using one-way analysis of variation (ANOVA) with Bonferroni's multiple comparison test. *n* = 15–27 **C.** Immunohistochemistry data. Staining intensities of PAICS and IMPDH2 in patient biopsies with BPH, PCa CRPC, hormone-naïve, short and long NHT were assessed. Intensities divided into four groups (negative = 0, weak = 1, moderate = 2 and strong = 3) and used for subsequent analysis. Statistical analysis was performed using one-way analysis of variation (ANOVA) with Bonferroni's multiple comparison test. *n* = 20–113 cores **D.** Representative images of the TMAs for PAICS (left) and IMPDH2 (right).

Taking together both the expression and ChIP approaches, we defined direct MYC regulated genes as genes that both mirrored changes in MYC levels in both directions and exhibited MYC binding in their respective promoter. Thus, PPAT, GART, PFAS, PAICS, ADSL and IMPDH2 are under the direct control of MYC in two metastatic PCa cell lines, as illustrated in Figure 2A.

### PAICS and IMPDH2 are overexpressed in two independent patient cohorts

Next we used recursive partitioning to determine whether at any expression threshold any of the genes in this pathway were predictive of post-operative BCR in the Taylor dataset. Two genes within the pathway, IMPDH2 (*p*-value = 0.036) and PFAS (*p*-value = 0.038), were significantly associated with time to BCR when overexpressed (Figure S3A). Based on overexpression in the Oncomine database and prognostic significance (Figure S1D and Figure S3A), we then went on to validate the expression of PAICS and IMPDH2 in two independent clinical cohorts using real-time PCR and immunohistochemistry (IHC).

First, we determined the relative mRNA expression levels of MYC and the two putative targets PAICS and IMPDH2 in a well-established patient cohort [23]. The expression of MYC was elevated in both PCa and CRPC patients (*p* < 0.05) with no significant difference between the two groups (Figure 2B). On the other hand, both PAICS and IMPDH2 RNA levels were significantly increased in CRPC patients only (*p* < 0.01 and *p* < 0.001, respectively) with no significant difference between benign hyperplasia (BPH) and PCa patients or PCa and CRPC (Figure 2B). The expression of MYC in CRPC samples was also tightly correlated with the expression of IMPDH2 (*R*^2^ = 0.504) but not with PAICS (*R*^2^ = 0.17) (Figure S3B).

Second, we examined the protein expression levels of PAICS and IMPDH2 using tissue microarrays (TMAs) in another patient cohort, previously used to assess a number of clinically relevant biomarkers, such as CAMKK2 or TAF1 [7, 28]. Examples of positive and negative controls for our staining can be found in the supplementary data (Figure S4). PAICS exhibited strong staining in less than 10% of the benign samples and in more than 45% of both PCa and CRPC patients, a highly significant increase (*p* < 0.0001 for both comparisons) (Figure 2C/D and S3C). Inversely, IMPDH2 showed high expression levels in 40% of benign samples and no significant increase in PCa patients. However, a significant increase (*p* < 0.05) could be observed in CRPC cases where more than 70% of the samples exhibited strong staining (Figure 2C/D and S3C). The TMA cohort also contained samples taken by repeat biopsy during treatment with neoadjuvant hormone therapy (NHT) over a 12 month period including pre-treatment samples. In contrast to androgen-regulated genes validated in this cohort, including CAMKK2 [7], neither PAICS nor IMPDH2 levels were significantly decreased by short (1–6 months) or prolonged (7–12 months) NHT (Figure 2C). Sustained high levels of expression make these enzymes interesting therapeutic targets and further support the androgen-independent regulation of their expression.

### Inhibition of IMPDH2 impairs proliferation of prostate cancer cell lines and shows additive effects with established anti-androgens

**Figure 3:**
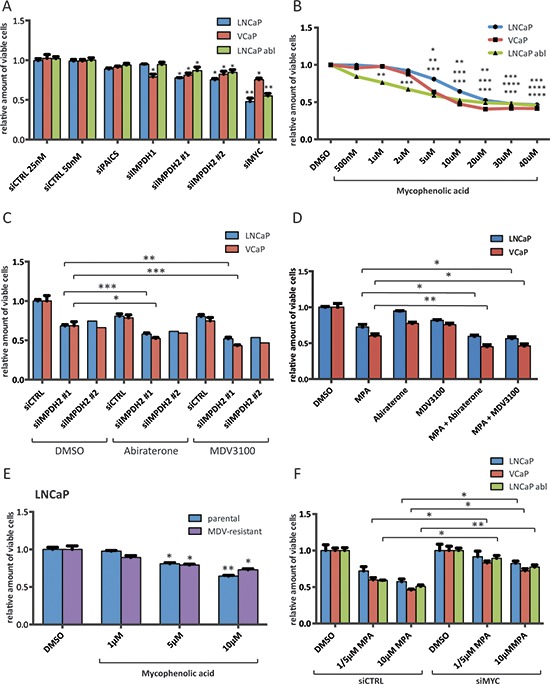
Inhibition of IMPDH2 impairs the proliferation of prostate cancer cells and shows additive effects with established anti-androgens **A.** Cell viability results of siRNA treated cells. Cells were transfected with 25 nM (control 25 nM, IMPDH1 and IMPDH2 #1) or 50 nM (control 50 nM, PAICS, IMPDH2 #2 and MYC) for 72 h and cell viability relative to siCTRL 50 nM was determined using a MTS-based assay. *n* = 2–4 **B.** Cell viability results of MPA treated cells. Cells were allowed to attach for 48 h prior to treatment with indicated doses of MPA for 72 h. Cell viability relative to vehicle control was determined using a MTS-based assay. *n* = 2–4 **C.** Cell viability results of siRNA and Abiraterone/MDV3100 treated cells. Cells were transfected with 25 nM IMPDH2 #1, 50 nM IMPDH2 #2 siRNA or equal amounts of siCTRL for 48 h. Following treatment with the indicated drugs for another 72 h, viability relative to DMSO and siCTRL was assessed using a MTS-based assay. Doses for LNCaP were 1 μM Abiraterone and 1 μM MDV3100, and for VCaP 1 μM Abiraterone and 100 nM MDV3100 *n* = 1–4 **D.** Cell viability results of MPA and Abiraterone/MDV3100 treated cells. Cells were allowed to attach for 48 h prior to treatment with indicated drug combinations for 72 h. Cell viability relative to vehicle control was determined using a MTS-based assay. Doses for LNCaP were 10 μM MPA, 1 μM Abiraterone and 1 μM MDV3100, and for VCaP 5 μM MPA, 1 μM Abiraterone and 100 nM MDV3100 *n* = 3–4 **E.** Cell viability results of MPA treated parental and MDV3100-resistant LNCaP. Cells were allowed to attach for 48 h prior to treatment with the indicated doses of MPA for 72 h. Cell viability relative to vehicle control was determined using a MTS-based assay. *n* = 4 **F.** Cell viability results of siRNA and MPA treated cells. Cells were transfected with 50 nM MYC or control siRNA for 48 h. Following treatment with the indicated doses of MPA (LNCaP 5 and 10 μM, VCaP 5 and 10 μM and LNCaP abl 1 and 10 μM) for another 72 h, viability was assessed using a MTS-based assay and normalized to the respective DMSO control. *n* = 3.

Next, to determine whether PAICS or IMPDH2 are essential for PCa cell proliferation, we applied siRNA-mediated knockdown and assessed the proliferation of LNCaP, VCaP and an derivative of LNCaP that has acquired resistance to androgen deprivation, LNCaP-abl [29] using a colourimetric-based assay (MTS). Whilst depleting these cells of PAICS had only a modest effect on proliferation, knockdown of IMPDH2 using two different siRNAs significantly (*p* < 0.05) reduced the number of viable cells in all three lines by about 15–20% (Figure 3A, knockdown efficacy was assessed in Figure S5A). We also included siRNA against IMPDH1, the other IMPDH isoform, and observed cell-line specific differences; LNCaP and LNCaP-abl were unaffected by IMPDH1 knockdown whilst VCaP exhibited effects comparable to IMPDH2 knockdown (Figure 3A). Intriguingly, a selective and uncompetitive inhibitor for IMPDH1 and 2, Mycophenolic acid (MPA) is a clinically approved immunosuppressant, commonly used to prevent organ transplant rejection [30]. MPA induced a dose-dependent inhibition of proliferation in all three lines (Figure 3B) and caused predominantly cytostatic effects as determined using a fluorescence-based assay to measure caspase cleavage in cultured cells over time (Figure S5C). For further experiments, we chose doses that reduced cell proliferation by about 20–30% (LNCaP 10 μM and VCaP 5 μM).

We have previously shown that the AR promotes glycolysis and anabolic metabolism [7]. Since purine biosynthesis appears to be a purely MYC-dependent pathway in androgen-responsive PCa cell lines without involvement of the AR, we hypothesized that combining either an inhibitor of androgen biosynthesis (Abiraterone) or a next-generation anti-androgen (Enzalutamide/MDV3100) with either siRNA against IMPDH2 or MPA would increase response to these drugs. Drug concentrations of Abiraterone and MDV3100 were chosen which gave maximally 20% reductions in viability when administered as single agents (Abiraterone: 1 μM for LNCaP and VCaP, MDV3100: 1 μM for LNCaP and 100 nM for VCaP). LNCaP-abl did not show a significant response to either Abiraterone or MDV3100 and thus were excluded from these experiments (data not shown). As hypothesized, combining siRNA against IMPDH2 with Abiraterone or MDV3100 had additive effects in both lines (Figure 3C). IMPDH2 knockdown alone induced a ~40% reduction in viability, either drug induced a ~20% reduction in viability, and combined we observed a ~60% reduction in viability (Figure 3C). We also performed these experiments using siRNA against siIMPDH1 (Figure S5B). As expected from the siRNA results alone (Figure 3A), we observed additive effects in VCaP but not LNCaP cells, suggesting that the expression and activity spectra of the IMPDH isoforms differ in both lines (Figure S5B). Combining MPA with Abiraterone or MDV3100 showed similar additive results (Figure 3D) and in all combinations the drugs were primarily cytostatic (Figure S5C). We also obtained a MDV3100-resistant LNCaP clone [31], and found that despite resistance to MDV3100 (20 μM), the clone remains equally responsive to MPA as the parental derivative (Figure 3E). Furthermore, we tested whether inhibition of IMPDH2 was dependent on MYC. Thus, we applied siRNA-mediated knockdown of MYC prior to treatment with two different doses of MPA (Figure 3F). We found that in all three lines, the efficacy of MPA was significantly (*p* < 0.05) reduced in a MYC-knockdown background (Figure 3F).

### MPA depletes cellular GTP levels and leads to nucleolar stress, p53 stabilization and downregulation of MYC

**Figure 4:**
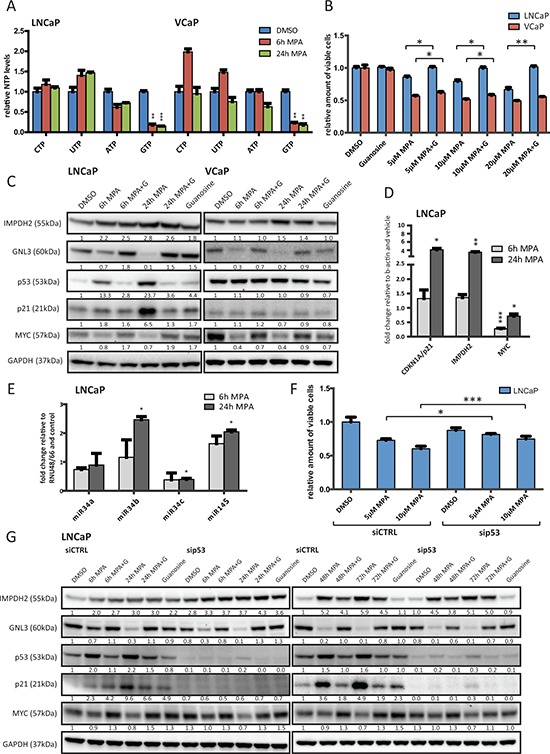
Inhibition of IMPDH2 leads to nucleolar instability, p53 activation and upregulation of MYC-targeting miRs **A.** HPLC results of cellular nucleoside triphosphate levels. LNCaP and VCaP cells were treated with 10 μM MPA for the indicated time points, lysed and subjected to HPLC to detect cellular levels of presented nucleoside triphosphates (NTP). Values were normalized to DMSO treated cells and protein content. *n* = 3 **B.** Cell viability results of MPA treated cells. Cells were allowed to attach for 48 h prior to treatment with indicated doses of MPA and guanosine (100 μM) for 72 h. Cell viability relative to vehicle control was determined using a MTS-based assay. *n* = 3 **C.** Western blot results of MPA treated PCa cells. LNCaP and VCaP cells were treated with 10 μM MPA and 100 μM guanosine (G) for the indicated time points. Protein extracts were harvested and subjected to Western blot analysis using the indicated primary antibodies. Protein levels were normalized to DMSO controls and GAPDH levels. **D.** Real-time PCR results. LNCaP cells were treated with 10 μM MPA for the indicated time points. Total RNA was isolated, reverse transcribed and used for qRT-PCR. *n* = 3 **E.** miRNA detection using real-time PCR of MPA treated LNCaP. Cells were treated with 10 μM MPA for the indicated time points prior to total RNA isolation using Trizol reagent and miR-detection using TaqMan assays. *n* = 2 **F.** Cell viability results of siRNA and MPA treated cells. Cells were transfected with 50 nM control or p53 siRNA for 48 h. Following treatment with the indicated drugs for another 72 h, viability was assessed using a MTS-based assay. *n* = 3 **G.** Western Blot results of siRNA and MPA treated LNCaP. Cells were transfected with 50 nM control or p53 siRNA for 48 h and then treated with 10 μM MPA and 100 μM guanosine (G) for the indicated time points. Protein lysates were harvested, separated by SDS-PAGE and blotted for the indicated proteins.

MPA was initially developed and clinically approved as an immunosuppressant to enhance transplant engraftment based on the increased nucleotide demands of expanding immune cell populations [30]. To confirm the selective effects of the drug on guanine nucleotide synthesis in LNCaP and VCaP cells, we analyzed the cellular nucleotide pools using high-pressure liquid chromatography (HPLC) after short (6 h) or prolonged (24 h) MPA treatment. We were able to confirm a significant reduction of cellular GTP pools to about 15–20% of the original levels, as expected from inhibiting an enzyme responsible for converting IMP to XMP, which in turn is converted to GMP (Figure 4A). Importantly, the levels of other nucleotide triphosphates remained largely unchanged. Furthermore, the cytostatic effects of MPA could be abolished in LNCaP and partially in VCaP cells by supplementing the cells with guanosine (100 μM), the final product of the guanine nucleotide biosynthetic pathway (Figure 4B).

GTP depletion has previously been shown to disrupt pre-rRNA synthesis and induce nucleolar stress [32]. In particular, the nucleolar protein guanine nucleotide-binding protein-like 3 (GNL3) has been reported to be destabilized when GTP biosynthesis is inhibited [33, 34]. Western blots of protein extracts harvested from MPA-treated LNCaP and VCaP cells on a time course up to 24 hours confirmed this stress response in both lines (reduction to approximately 10–20% after 24 h treatment), which could be rescued by adding guanosine (Figure 4C). Nucleolar stress has been shown to stabilize p53 and trigger cell cycle arrest [35–37]. We therefore blotted for p53 and found that MPA treatment led to a significant increase in p53 levels in the wild-type line LNCaP (approximately 10 to 20-fold), whilst failing to do so in the p53-mutant VCaP line (Figure 4C). This was corroborated by an upregulation of the direct p53 target p21 (CDKN1A) by about 5 to 6-fold, both in protein and mRNA levels (Figure 4C and 4D, respectively). We also observed a previously reported compensatory increase in IMPDH2 mRNA and protein (approximately 4-fold and 2 to 3-fold, respectively), albeit without a recovery in GTP levels (Figure 4C, 4D and 4A). In addition, MPA treatment led to a feedback reduction in MYC expression in both the protein (30–60% reduction at 24 h) and transcript levels (maximum 70% reduction at 6 h) (Figure 4C and 4D, respectively).

This implied an inhibition of either MYC transcription or mRNA stability/processing. A number of microRNAs have recently been reported to repress MYC expression and function as tumour suppressors, including the let7 and miR-34 clusters as well as miR-145 [38–40]. Some of these are also known to be p53 targets and we selected a subset with both characteristics to test whether MPA treatment also increased the expression of these miRNAs. Confirming our hypothesis, 24 h MPA treatment of the p53 wild-type cell line LNCaP significantly increased miR-34b expression approximately 2.5-fold and miR-145 expression approximately 2-fold while at the same time suppressing miR-34c by about 50% (Figure 4E). In contrast, the expression of miR-34a was not significantly altered. These miRNAs could not be detected in VCaP cells (data not shown), suggesting that other mechanisms might contribute to the observed downregulation of MYC in this line.

To assess the role of p53 in the wild type line LNCaP, we knocked down its expression using siRNA and treated the cells with MPA (Figure 4F/G). p53 knockout partially restored the proliferation of LNCaP cells (by approximately 10–15%), suggesting that p53 and putatively p21 are at least partially responsible for the observed reduction in proliferation (Figure 4F). We also treated the cells with MPA on an extended time course (6–72 h) and blotted for IMPDH2, GNL3, p53, p21 and MYC (Figure 4G). Interestingly, no major changes in the protein levels of GNL3 and MYC could be observed, which suggested that the effects of MPA on these proteins were independent of p53. This was further corroborated by the observation that hydroxyurea, a known ribonucleotide reductase inhibitor and activator of p53, had no effect on GNL3 and only a modest effect on MYC levels (Figure S5D).

In conclusion, we find that IMPDH2 expression is elevated in PCa and that inhibition has the feedback capacity to reduce the expression of MYC and to increase the sensitivity of PCa cells to anti-androgens and androgen synthesis inhibitors.

## DISCUSSION

The oncogenic transcription factor MYC is a complex transcription factor and even after decades of research, its exact functions in PCa initiation and progression remain unclear. Previous unbiased approaches in other cell types, such as embryonic stem cells and human B-cells, revealed MYC to be the driver of anabolic metabolism and biomass accumulation through the regulation of amino acid metabolism, ribosome biogenesis and purine metabolism [14, 15, 41]. In PCa, the amplification of the MYC locus on 8q24 is predictive of poor prognosis [42, 43]. Additional increased MYC expression predicts poor outcome and overexpression of MYC in PCa cell lines and mouse models promotes castrate-resistant and mTOR inhibitor-resistant growth, and alters the expression levels of other hormone-receptors, such as estrogen receptor alpha (ERa) [10, 44–46]. However, so far identification of MYC-dependent genes mediating these effects in PCa has been limited to a study reconfirming the contribution of MYC to the expression of ribosomal and nucleolar genes [17]. For the first time, our work combined an *in vitro* approach using an inducible MYC overexpression system in LNCaP cells and *in silico* analysis of a clinical dataset in order to define clinically relevant MYC transcriptional networks in PCa (Figure 1). We reconfirmed the MYC-dependent expression of nucleolar and ribosomal genes, but progressed beyond this to identify a metabolic pathway, which alters the response to AR-targeted therapies, the *de novo* purine biosynthesis pathway. This pathway, alongside ribosome and nucleolar biogenesis, can be regarded as supporting processes to sustain elevated rates of transcription, replication and cell division in transformed cells. Interestingly, the genes in the purine biosynthesis pathway are not androgen regulated, but rather MYC dependent (Figure S1F/G and Figure 1C–1F), which is intriguing in the light of a recent report highlighting AR as a driver of several anabolic processes, including the pentose phosphate shunt pathway, which feeds metabolites into the purine biosynthesis pathway [7]. In this study, however, the expression levels of the enzymes themselves were not reported to be AR regulated.

We then went on to address the question whether enzymes within the pathway might serve as cancer biomarkers. Neither PAICS nor IMPDH2 have been evaluated as potential biomarkers in PCa. However, IMPDH2 has been tested as a potential biomarker using IHC in colorectal cancer and appears to be a promising target for both detection and treatment [47]. Our findings suggest potential for IMPDH2 as a biomarker in PCa since a significant increase in mRNA levels could only be observed in CRPC and not in BPH or localized PCa samples (Figure 2B). Furthermore, CRPC cases exhibited a significant increase in IHC staining when compared to BPH samples (Figure 2C).

On the other hand, PAICS has not been assessed as a potential biomarker in any cancer previously. Similar to IMPDH2, PAICS mRNA levels were only significantly increased in CRPC but not in PCa (Figure 2B). Strong IHC staining for PAICS, however, occurred almost exclusively in PCa or CRPC cases and almost 50% of the BPH cases exhibited only weak staining (Figure 2C). Recent work highlights the metabolite SAICAR (the product of the reaction catalyzed by PAICS) as a regulator of pyruvate kinase and describes its growth-promotion functions in glucose-limited conditions - conditions that often occur in the nutrient-limited environment of solid tumours [48]. PAICS appears to be regulated by MYC in other cancer types, which further emphasizes the important roles this enzyme may play in cancer [49]. Our study is the first to assess it as a potential biomarker in any cancer and we find that its expression is significantly increased at the transcript and protein level in two cohorts, meriting future studies to assess it as a surrogate marker for the pathway as a whole.

Next, we determined whether IMPDH2 or PAICS constitute important metabolic enzymes for maintaining the proliferation of androgen-responsive and -independent PCa cell lines (Figure 3). Whilst IMPDH2 knockdown using two different siRNAs significantly reduced cell viability in three different cell lines, PAICS knockdown had no effect (Figure 3A). This, however, may relate to the differing contributions of these enzymes to metabolite production as PAICS is a multifunctional enzyme in the *de novo* purine biosynthesis pathway but not reported to be rate limiting [50]. By contrast, IMPDH2 is a rate-limiting enzyme for guanine nucleotide biosynthesis and we hypothesized that the growth reduction observed by IMPDH2 knockdown might reflect depletion of guanine nucleotides. Inhibitors of IMPDH1/2 have been available for decades, initially to limit immune responses to transplant surgery, with the best characterized example being Mycophenolic acid (MPA) [30]. Treatment with this drug led to > 80% reduction in GTP levels (Figure 4A) and to significant reduction in cell viability, which could be rescued completely in LNCaP and partially in VCaP cells by adding guanosine, the final product of the guanine nucleotide biosynthetic pathway (Figure 3B and Figure 4B). In contrast to LNCaP cells, VCaP cells are sensitive to siRNA-mediated knockdown of IMPDH1, a second isoform of the IMPDH family (Figure 3A). MPA is known to target both isoforms and the difference in sensitivity to IMPDH1 knockdown may contribute to the inability of guanosine to rescue MPA-treated VCaP completely at equivalent concentrations to those, which effectively rescued LNCaP (Figure 4B).

Next, we combined an anti-androgen (MDV3100) and an androgen synthesis inhibitor (Abiraterone) with MPA or siRNA against IMPDH2 and observed an additive reduction in cell viability (Figure 3C and 3D), which further highlights the AR-independence of this pathway. We also observed an additive reduction in viability of IMPDH1 knockdown in combination with Abiraterone and MDV3100, albeit only in VCaP cells (Figure S5B). This suggests cell line specific differences in the expression and activity spectra of the IMPDH isoforms. Furthermore, a MDV3100 resistant LNCaP clone remained sensitive to MPA (Figure 3E). This is particularly intriguing as MDV3100-resistance has recently been observed in late-stage CRPC patients [51]. We also assessed the dependency of MPA inhibition on MYC expression and found that MYC knockdown significantly reduced the efficacy of MPA underscoring the tight relationship between MYC and the response to a drug targeting a MYC-dependent enzyme (Figure 3F).

Among other core cellular processes, GTP has previously been reported to be critical for regulating nucleolar stability and assembly, in part through binding by the nucleolar protein GNL3 [52]. It has been shown that GTP depletion triggers the degradation of GNL3 and other nucleolar components, thereby leading to nucleolar stress [33]. We confirmed that MPA treatment induced these effects in PCa cells, as well as a previously reported feedback stabilization of the target enzyme, IMPDH2, due to the formation of a stable complex with MPA [53] (Figure 4C). Importantly, increased protein levels of IMPDH2 failed to restore GTP levels (Figure 4A). Although not explicitly tested for in this setting, it is conceivable that MPA treatment induces widespread changes in cellular nucleotide metabolism. IMP, a substrate for IMPDH1/2, is also an important substrate for other enzymes, such as ADSSL1, which synthesizes AMP (Figure 2A). Consequently, IMP is at a branching point in the purine biosynthesis pathway and the conversion of IMP to either GMP or AMP is energy-dependent. The energy source, however, is different for each product and reciprocal, i.e. ATP as an energy source drives GMP synthesis and conversely, GTP drives AMP production. Thus, the accumulation of excess ATP favours GMP production. Furthermore, the nucleotide salvage pathway can sustain IMP levels through the activity of the enzyme hypoxanthine-guanine phosphoribosyltransferase (HGPRT) [54]. Consequently, MPA treatment may induce compensatory effects, not sufficient to offset nucleolar stress where there is a strong guanine nucleotide dependency, but perhaps to upregulate other IMP converting enzymes (e.g. ADSSL1), as observed in response to MYC knockdown (Figure 1D) or upregulation of enzymes of the purine salvage pathway (e.g. HGPRT) to salvage the scarce guanosine [54].

Nucleolar stress has pleiotropic tumour suppressive effects depending on cell type and genetic background. One major effect can be on p53 stability and activity, with stress induction leading to the stabilization of p53 by inhibiting the MDM2-p53 complex [32]. We found that MPA treatment stabilized p53 in PCa cells in a wild-type setting (LNCaP) whilst failing to have a significant impact on p53 levels in a mutant line (VCaP) (Figure 4C). This was also observed for a well-characterized target gene of p53, the cyclin-dependent kinase inhibitor p21 (CDKN1A) (Figure 4C and 4D). Interestingly, in both cell lines MYC levels were significantly reduced upon MPA treatment at both the transcript and protein level (Figure 4C and 4D). It has previously been shown that MYC levels in PCa cells can be reduced through the activity of the miR-34 cluster and miR-145 [39, 40]. Since these miRs are also p53-regulated [55, 56], we tested whether they were overexpressed in response to MPA treatment. Two miRs, miR-34b and miR-145, were increased in expression in the LNCaP cell line (Figure 4E).

In order to assess the contribution of p53 to these changes, we knocked down its expression using siRNA in the LNCaP cell line. Knockdown of p53 rendered the LNCaP cell line less sensitive to the cytostatic effects of MPA (Figure 4F). Strikingly, this was not due to changes in GNL3 or MYC protein levels, as assessed by western blotting on an extended timecourse from 6–72 h treatment (Figure 4G). This suggests that while p53 appears to be, at least partially, responsible for the cytostatic effects of MPA in LNCaP, nucleolar stress and downregulation of MYC occur independently of p53. This is in line with our observations in the p53-mutant cell line VCaP where both GNL3 and MYC are downregulated in the absence of a significant increase in p53 or p21 (Figure 4C). Conversely, hydroxyurea, a known ribonucleotide reductase inhibitor and activator of p53, had no effect on GNL3 and only a modest effect on MYC levels (Figure S5D), suggesting the induction of nucleolar stress is specific to MPA.

Strikingly, several other mechanisms of MYC-suppression have been discovered in various cell types and these might also contribute to the observed downregulation of MYC in our experiments. For example, the ribosomal proteins RPL5 and RPL11, which are responsible for the inhibition of the MDM2-p53 complex and concomitant stabilization of p53, have also been shown to suppress MYC directly both on transcript and protein level in lung cancer cells [57, 58]. This might be one possible explanation for why VCaP cells, which harbour a p53 mutation, also respond to MPA. However, the exact functional impact of the p53 mutation in VCaP has not been explored yet.

In this paper, we were able to show in the LNCaP cell line strong associations between MPA treatment, p53 stabilisation and the increased expression of known p53-driven oncosuppressive microRNAs, miR-34b and miR-145, which have previously been reported to target MYC. Therapeutically, these miRs are particularly interesting in the light of recently published novel cancer therapy approaches, which involve the restitution of tumour suppressive miRs, including miR-145, through viral or nanoparticle delivery of expression vectors [59, 60]. The challenge is to translate these novel therapeutics into patients. In contrast to this approach, MPA is already a commonly prescribed immunosuppressant and well-characterised in a clinical setting. Current treatment options for CRPC include anti-mitotic chemotherapeutics (Docetaxel and Cabazitaxel), AR-targeting agents (Abiraterone and MDV3100) and notably the immunosuppressant Prednisone. A very recent trial of prednisone in combination with Abiraterone showed a significant survival benefit for the combination in men with chemotherapy-naive castration-resistant prostate cancer [61]. Our study is the first to suggest a second, clinically approved immunosuppressant as a putative treatment option for late-stage CRPC. However, further validation in preclinical models of PCa will be needed to assess the suitability of MPA treatment in patients [62].

In conclusion, our study highlights the potential to exploit purine biosynthesis and specifically the activity of IMPDH2 to diminish the expression and activity of MYC and enhance response to clinically approved PCa drugs. Whilst MPA has long been clinically approved, inhibitors with greater selectivity for IMPDH2 are under development [63] and it remains to be seen whether these drugs will provide greater efficacy, particularly in PCa. New IMPDH inhibitors will certainly provide greater opportunities for combination trials in the near future and the potential to do so is also enhanced by the existence of assays to measure GTP levels in peripheral blood mononuclear cells and the levels of IMPDH2 in serum [64–66]. This might potentially be relevant as surrogate response and treatment stratification markers but needs to be further assessed in PCa cohorts. Finally, as the relationship between transcription and metabolism in PCa is more extensively explored, so additional clinically relevant feedback loops will be uncovered and provide new targets for intervention.

## MATERIALS AND METHODS

A more detailed version of the materials and methods used can be found in the supplemental material.

### Immunohistochemistry

This study was done on a total of 194 PCa specimens obtained from Vancouver Prostate Centre Tissue Bank. The H&E slides were reviewed and the desired areas were marked on them and their correspondent paraffin blocks. 3 TMAs were manually constructed (Beecher Instruments, MD, USA) by punching duplicate cores of 1 mm for each sample. All the specimen were from radical prostatectomy except 12 CRPC samples that obtained from transurethral resection of prostate (TURP).

Immunohistochemical staining was conducted by Ventana autostainer model Discover XT^TM^ (Ventana Medical System, Tuscan, Arizona) with enzyme labeled biotin streptavidin system and solvent resistant Red Map kit by using 1:600 of PAICS rabbit polyclonal antibody (Sigma, HPA035895) and 1:2,000 concentrations of IMPDH2 rabbit monoclonal antibody (Epitomics, 5814–1), respectively.

The antibodies were validated in the following manner.

PAICS: Using Protein Atlas as our reference, we stained Ovarian Cancer and Normal Liver as our positive controls. Prostate Stromal cells were our negative controls.

http://www.proteinatlas.org/ENSG00000128050-PAICS/cancer

IMPDH2: Using Protein Atlas as our reference, we stained Ovarian Cancer and Benign atrophic Testis as our positive control. Prostate Stromal cells were our negative controls.

http://www.proteinatlas.org/ENSG00000178035-IMPDH2/cancer

Representative images of positive and negative controls can be found in Figure S4.

Values on a four-point scale were assigned to each immunostain. Descriptively, 0 represents no staining by any tumor cells, 1 represents a faint or focal, questionably present stain, 2 represents a stain of convincing intensity in a minority of cells and 3 a stain of convincing intensity in a majority of cells.

### Quantitative real-time PCR (qRT-PCR)

Samples were run in duplicates and transcript levels were normalized to vehicle controls and the expression levels of β-actin using the 2^ddCt method. A list of primers used for the real-time PCR experiments can be found in Figure S2B.

Real-time PCR on clinical samples was performed as previously described [23]. For a detailed list of primer pairs used, see Figure S2B.

The quantification of miRNA levels was performed according to the TaqMan Micro-RNA Assays protocol (Applied Biosystems). Briefly, RNA was reverse transcribed with miR–34a, –34b, –34c and –145 specific primers (TaqMan Assay nos. 000426, 002102, 000428, and 002278 respectively) and samples were run in quadruplicates. Transcript levels were normalized to vehicle controls and the expression levels of the geometric mean of RNU48 and RNU66 using the 2^ddCt method.

### Chromatin immunoprecipitation (ChIP)

ChIP was performed using the Human MYC ExactaChIP Chromatin IP kit (R&D, ECP3696) and 1% of total chromatin was taken as input control prior to overnight incubation with the antibody. ChIP-qPCR was perfomed using the SYBR green master mix and same amplification conditions as for qRT-PCR. Results are being displayed as ‘% of input’ using the formula 2^(ct(Input)-ct(antibody)). For a detailed list of primer pairs used, see Figure S2B.

### siRNA transfection

The following siRNAs were used: ON-TARGETplus Non-Targeting Pool (Thermo Scientific, D-001810–10), ON-TARGETplus Human MYC SMARTpool (Thermo Scientific, L-003282–02), ON-TARGETplus Human PAICS SMARTpool (Thermo Scientific, L-003980–00), siIMPDH2 #1: siIMPDH2 Silencer Select (Ambion, 106309), siIMPDH2 #2: ON-TARGETplus Human IMPDH2 SMARTpool (Thermo Scientific, L-004330–00) and Human p53 SMARTpool (Thermo Scientific, L-003329–00).

### Western blot analysis

Primary antibodies used were PAICS (Sigma, HPA035895), IMPDH2 (Sigma, HPA001400), MYC (Abcam, ab32072), AR (Santa Cruz, sc-816), p53 (Santa Cruz, sc-126), GNL3 (R&D, AF1638), KLK3 (Dako, D0487), b-actin-HRP (Cell Signaling, 5125) and GAPDH (Cell Signaling, 2118). Secondary HRP-conjugated anti-rabbit and anti-mouse were purchased from Dako (P0448 and P0447, respectively). Densitometry analysis was performed using the freely available software ImageJ and protein levels normalized to vehicle controls and the indicated loading control (b-actin or GAPDH).

### Statistics

Unless stated otherwise, mean values with standard error of the mean (SEM) are displayed and significance was confirmed using paired two-tailed Student's *t*-test. *p < 0.05, **p < 0.01, ***p < 0.001, ****p < 0.0001

## SUPPLEMENTARY DATA








