# Supplementary material for: Myc-dependent purine biosynthesis affects nucleolar stress and therapy response in prostate cancer
Source: Oncotarget. 2015 Mar 30;6(14):12587–602. doi: 10.18632/oncotarget.3494 (PMC4494960; doi:10.18632/oncotarget.3494)
Supplement: Supplementary file 1 [file oncotarget-06-12587-s001.pdf]

SUPPLEMENTARY DATA

Cell culture

LNCaP and VCaP cells (ATCC) were cultured at 37°C and 5% CO2 in RPMI1640 or DMEM (Gibco, 21875 and 41966), respectively, containing 10% fetal bovine serum (FBS) (Gibco, 10500). LNCaP abl cells were cultured in RMPI1640 containing 10% charcoal-stripped FBS (Gibco, 12676029). For hormone starvation, LNCaP and VCaP were washed once with PBS (Gibco, 10010) and cultured in phenol red-free RMPI1640 or DMEM (Gibco, 11835063 and 31053028), respectively, containing 10% charcoal-stripped FBS (Gibco, 12676029) for 72 h before starting the experiment. LNCaP MYC cells were cultured analogous to the parental cell line with the addition of 2 µg/ml Puromycin and 200 µg/ml G418 (Gibco, 10131019) for plasmid maintenance.

For viability assays, the amount of viable cells was determined using Cell Aequous solution MTS reagent (Promega, G3581) following the manufacturer's recommendations.

Chemicals

Puromycin (Gibco, A1113803), G418 (Gibco, 10131019), R1881 (Sigma, R0908), Abiraterone (SelleckBio, S2246), MDV3100 (SelleckBio, S1250), Mycophenolic acid (Sigma, M3536), Doxycycline (Sigma, 44577), 2-Deoxy-D-Glucose (Sigma, D3179) and Metformin (Sigma, M0605000) were dissolved and stored according to manufacturer's recommendations. All other chemicals used were purchased from Sigma if not stated otherwise.

Hydroxyurea was a kind gift of Dr. Hilde Nielsen (The Biotechnology Centre, Oslo, Norway).

RNA isolation and processing for microarrays

Total RNA was isolated using the Qiagen RNeasy kit (Qiagen, 74106) following the manufacturer's recommendations. RNA concentration and purity was measured using a NanoDrop instrument (Thermo Scientific).

For microRNA-profiling, total RNA was isolated using Trizol reagent (Life technologies, 15596026) following the manufacturer's recommendations.

For microarray analysis, RNA integrity was confirmed using a 2100 Bioanalyzer (Agilent) and Total RNA Nano Chip (Agilent, 5067–1511). 500 ng RNA was reverse transcribed and Biotin-labeled using the TotalPrep-96 RNA Amplification kit (Illumina, 4393543) following the manufacturer's recommendations. Resuspended cRNA samples were hybridized onto Human HT-12 Expression BeadChips (Illumina, BD-103–0204). Missing probes were imputed using Illumina's GenomeStudio Gene Expression Module.

Microrarray analysis

The imputed probe datasets were analyzed using the freely available J-Express 2012 software (http://jexpress.bioinfo.no/site/). The raw data was quantile normalized and log2 transformed prior to analysis. Differential expression analysis was performed using the grouped triplicate experiments and Rank product analysis. Probes with a *q*-value of < 0.05 were considered significantly up- or downregulated. For hierarchical clustering using complete linkage and Pearson correlation, differentially expressed probes were merged and high level mean and variance normalized.

Gene expression data published in Taylor et al., were downloaded from the NCBI GEO data repository (accession number: GSE21034) and analyzed using J-Express.

siRNA transfection

LNCaP and VCaP cells were 'reverse' transfected. For 6-well plates, 500 µl Opti-MEM (Invitrogen, 11058–021), 5 µl Lipofectamine RNAimax (Invitrogen, 13778–150) and 3.75–7.5 µl siRNA (25/50 nM) were mixed in the well and incubated at RT for 20 min. Cells were trypsinated and resuspended at 1×10^5/ml (LNCaP and LNCaP abl) or 3×10^5/ml (VCaP) in regular serum-containing medium and 2.5 ml were added to the siRNA complexes. For other plate formats, volumes and cell numbers were adjusted accordingly.

Reverse transcription and quantitative real-time PCR (qRT-PCR)

500 ng to 1 µg total RNA was reverse transcribed using the SuperScript VILO kit (Applied Biosystems, 11754) following the manufacturer's recommendations. qRT-PCR was performed using SYBR green master mix (Applied Biosystems, 4385612). Amplification was performed in duplicate series using the ABI 7900HT FAST Sequence Detection System (Applied Biosystems) with the following cycling conditions, 50°C for 2 min, 95°C for 10 min, 40 cycles of 95°C for 15s and 60°C for 60s. Transcript levels were normalized to vehicle controls and the expression levels of beta-actin using the 2^ddCt method.

Western blot analysis

Cells were trypsinized and washed with cold PBS prior to resuspension in RIPA lysis buffer (30 mM Tris-HCl, 150 mM NaCl, 1mM EDTA, 0.5% NP40, 0.1% Na-Deoxycholate, 0.1% SDS, pH 7.4) supplemented with protease inhibitors (Roche, 11873580001), rotated at 4°C for 10 min and sonicated in a Bioruptor NextGen (Diagenode) at maximum power for ten cycles of 30s ON, 30s OFF to break nuclei and other cellular structures. Lysates were centrifuged for 10 min at 18,000 g and 4°C and the supernatant transferred to a new tube. Protein concentration was determined using a BCA assay (Pierce, 23227) and equalized with RIPA buffer. Extracts were mixed with LDS NuPAGE buffer (Life technologies, NP0008) and Sample Reducing Agent (Life technologies, NP0009) and denatured for 10 min at 70°C. Equal amounts were loaded onto 4–12% gradient Bis-Tris NuPAGE gels (Life technologies, NP0323). Separated proteins were wet-blotted (25 mM Tris-Base, 192 mM glycine, 20% methanol, 0.01% SDS, pH 9.2) to methanol-activated 0.45 µm PVDF membranes (Millipore, IPVH00010) for 60 min at 30 V. Membranes were blocked in 5% BSA (Sigma, A2153) in TBS with 0.1% Tween-20 (Sigma, P5927) for 1 h prior to overnight incubation with appropriate concentrations of primary antibodies. The next day, membranes were washed with TBS with 0.1% Tween-20 and incubated with appropriate secondary antibody for 1 h at room temperature. After washing with TBS with 0.1% Tween-20, membranes were developed using the Novex ECL Reagent kit (Life technologies, WP20005) or a super-sensitive HRP substrate (Rockland, FEMTOMAX-110). Primary antibodies used were PAICS (Sigma, HPA035895), IMPDH2 (Sigma, HPA001400), MYC (Abcam, ab32072), AR (Santa Cruz, sc-816), p53 (Santa Cruz, sc-126), GNL3 (R&D, AF1638), KLK3 (Dako, D0487), b-actin-HRP (Cell Signaling, 5125) and GAPDH (Cell Signaling, 2118). Secondary HRP-conjugated anti-rabbit and anti-mouse were purchased from Dako (P0448 and P0447, respectively). Densitometry analysis was performed using the freely available software ImageJ and protein levels normalized to the respective loading control.

Identification of putative MYC binding sites and primer design

To identify candidate consensus MYC binding sites associated with purine biosynthesis enzymes, we downloaded ENCODE datasets from the National Center for Biotechnology Information (NCBI) data repository (http://www.ncbi.nlm.nih.gov/geo/query/acc.cgi) for six cell lines: lymphoblastoid cells - GM12878 (GSM822290); embryonic stem cells - H1-hESC (GSM822274); hepatocytes - HepG2 (GSM822291); cervical cancer cells - HeLa-S3 (GSM935320); endothelial cells - HUVEC (GSM822298) and myeloid leukemia cells - K562 (GSM935516). We identified overlapping MYC consensus binding sites in a minimum of three cancer cell lines lying at the transcription start sites (TSS) of every gene in the pathway. Approximate primer sites are shown in Figure S2A (black rectangle) and sequences can be found in Figure S2B.

Chromatin immunoprecipitation (ChIP)

ChIP was performed using the Human MYC ExactaChIP Chromatin IP kit (R&D, ECP3696) with slight modifications to the manufacturer's protocol. Briefly, LNCaP or VCaP cells were seeded in charcoal-stripped medium in 150 mm dishes for 72 h prior to 4 h stimulation with 1 nM R1881 or EtOH control and crosslinking with 1% formaldehyde (Sigma, F8775). After scraping and cell lysis, chromatin was sheared to an average fragment size of 200–500 bp using a Bioruptor NextGen (Diagenode), cleared and diluted. Prior to overnight incubation with 5 µg MYC/control IgG antibody, 1% of total chromatin was taken as input control. After harvesting the antibody-DNA complexes on a 4°C rotator for 1 h using 50 µl magnetic Streptavidin beads (R&D, MAG999), the DNA was eluted and reverse crosslinked for 16–20 h shaking at 65°C using 200 µl of a 1% SDS in NaHCO3 solution. Subsequently, the DNA was purified using phenol-chloroform-isoamylalcohol extraction and reverse phase-lock tubes (5Prime, 2302830), precipitated with EtOH abs., washed with 80% EtOH and finally resuspended in 60 µl Tris-HCl (pH 8.0). ChIP qPCR was performed using the SYBR green master mix and same amplification conditions as mentioned above. Results are being displayed as '% of input' using the formula 2^(ct(Input)-ct(antibody)). For a detailed list of primer pairs used, see Figure S2.

Recursive partitioning

To identify statistically significant biochemical recurrence courses, recursive partitioning was performed on a single gene expression profile using the 'party' package from CRAN (Han et al, 2012) with accompanying biochemical recurrence data taken from (Huang et al, 2008). Kaplan Meier plots of the risk of biochemical recurrence were produced using the 'survival' package and *p*-values from the log rank test were corrected using the Bonferroni correction method.

Fluorescent-based caspase cleavage assay

Appropriate amounts of LNCaP or VCaP cells were seeded in 384-well plates and allowed to attach for 48 h at which point they received drug treatment (12 wells

per condition). Induction of apoptosis was monitored using the CellPlayer 96-well Caspase-3/7 reagent (Essen Bioscience, 4440) at a final concentration of 1:5,000 on the Incucyte FLR instrument (Essen Bioscience). Phase contrast and fluorescence pictures were taken every two hours for a total of 96 h. Analysis was performed using the inbuilt object counting algorithm.

NTP measurements

LNCaP and VCaP cells were seeded in 100 mm dishes and allowed to attach for 48 h at which point the received fresh drug-containing medium. For every condition, a duplicate plate was included for protein normalization. Cells were washed once with a 150 mM NaCl solution, scraped and immersed in 600 µl of ice-cold extraction solution (15% w/v trichloroacetic acid, 15 mM $MgCl_2$) and frozen in liquid nitrogen. The samples were vortexed for 30s and incubated for 10 min at 4°C. Supernatants were collected after centrifugation at 20,000 g for 1 min, and added to 800 µl of ice-cold extraction mixture consisting of 10 ml of Freon (1, 1, 2-trichlorotrifluoroethane, Aldrich, Sigma-Aldrich Sweden AB > 99%) and 2.8 ml of trioctylamine (Sigma-Aldrich Sweden AB, 98%). The samples were vortexed and centrifuged for 1 min at 20,000 g. The aqueous phase was collected and added to 700 µl of the same ice-cold extraction mixture. The samples were vortexed as above. 70 µl of the aqueous phase containing nucleoside triphosphates (NTP) was adjusted to pH 3.4 with 6 M HCl, separated on a Partisphere SAX HPLC column (125 mm × 4.6 mm, Hichrome, United Kingdom) under isocratic elution with 0.35 M potassium phosphate buffer (pH 3.4, 2.5% v/v acetonitrile) and quantified using a LaChrom Elite® HPLC system (VWR International).

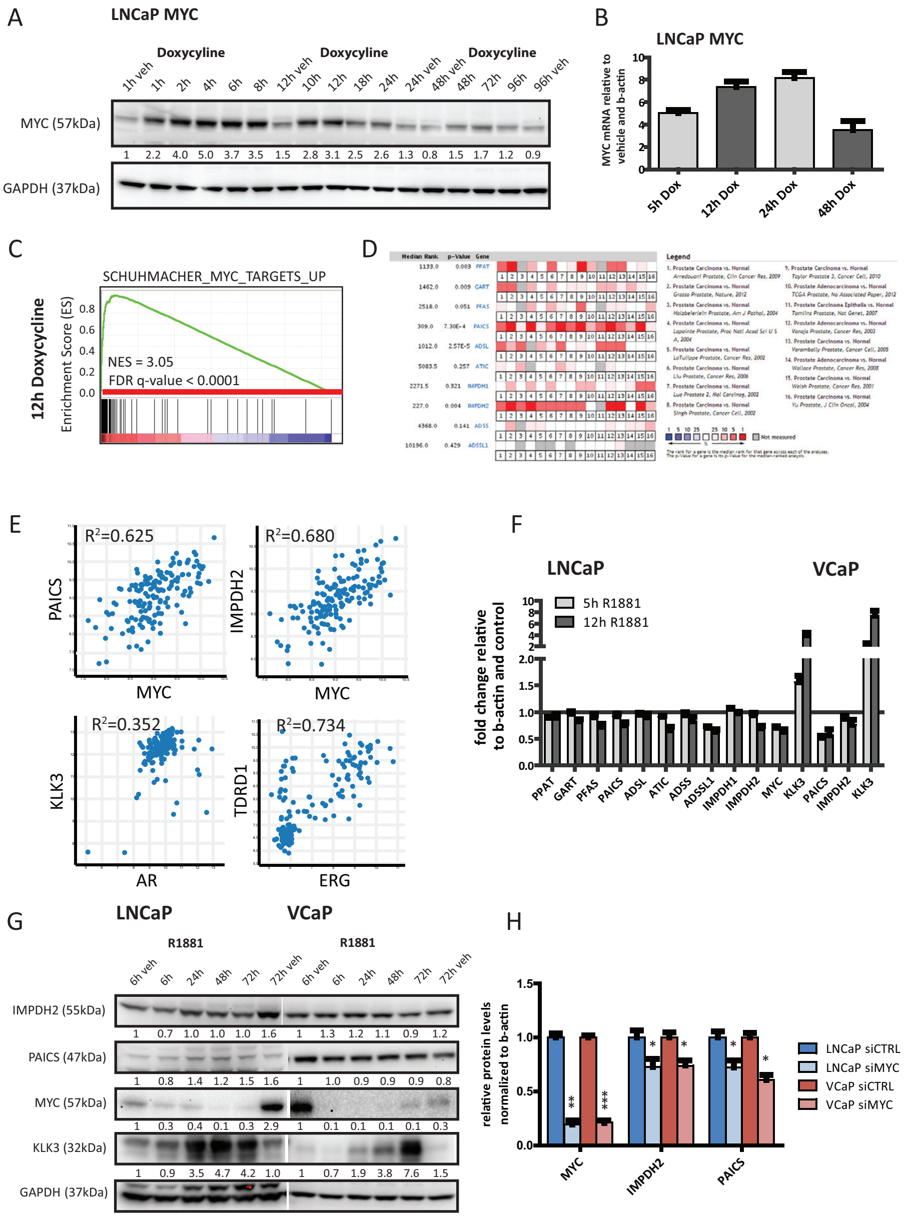

Supplementary Figure S1: (A-B) LNCaP MYC cells were treated with 2 µg/ml Doxycyline for the indicated timepoints to induce MYC overexpression. **A.** Western blot analysis of LNCaP MYC-overexpressing cells. Protein lysates were harvested, separated by SDS-PAGE and blotted for the indicated proteins. Protein levels were normalized to 1 h vehicle control and GAPDH. **B.** Real-time PCR results of LNCaP MYC-overexpressing cells. Total RNA was isolated, reverse transcribed and used for qRT-PCR. mRNA levels were normalized to b-actin levels and vehicle treated control. *n* = 2–3 **C.** The top upregulated gene set using Gene Set Enrichment Analysis (GSEA) of 12 h Doxycycline-treated LNCaP MYC is shown **D.** Meta-analysis of 16 clinical PCa vs. normal datasets using the Oncomine browser is shown. **E.** Correlation coefficient curve. The log2 normalized expression values of the indicated gene combinations were plotted and a correlation coefficient was calculated to assess coexpression of the respective genes (based on Taylor *et al*.). **(F-G)** LNCaP and VCaP cells were hormone-starved for 72 h prior to stimulation with 1 nM R1881 for the indicated timepoints. **F.** Real-time PCR results of LNCaP and VCaP cells. Total RNA was isolated, reverse transcribed and used for qRT-PCR. mRNA levels were normalized to b-actin levels and vehicle treated control. *n* = 3 **G.** Western blot analysis of 72 h hormone-starved LNCaP and VCaP cells treated with vehicle or 1 nM of the synthetic androgen R1881. Protein lysates were harvested, separated by SDS-PAGE and blotted for the indicated proteins. Protein levels were normalized to 6 h vehicle control and GAPDH. **H.** Cells were transfected with 50 nM of control or MYC siRNA for 72 h. Densitometry analysis of four biological replicates of the representative images in Figure 1E is displayed.

A

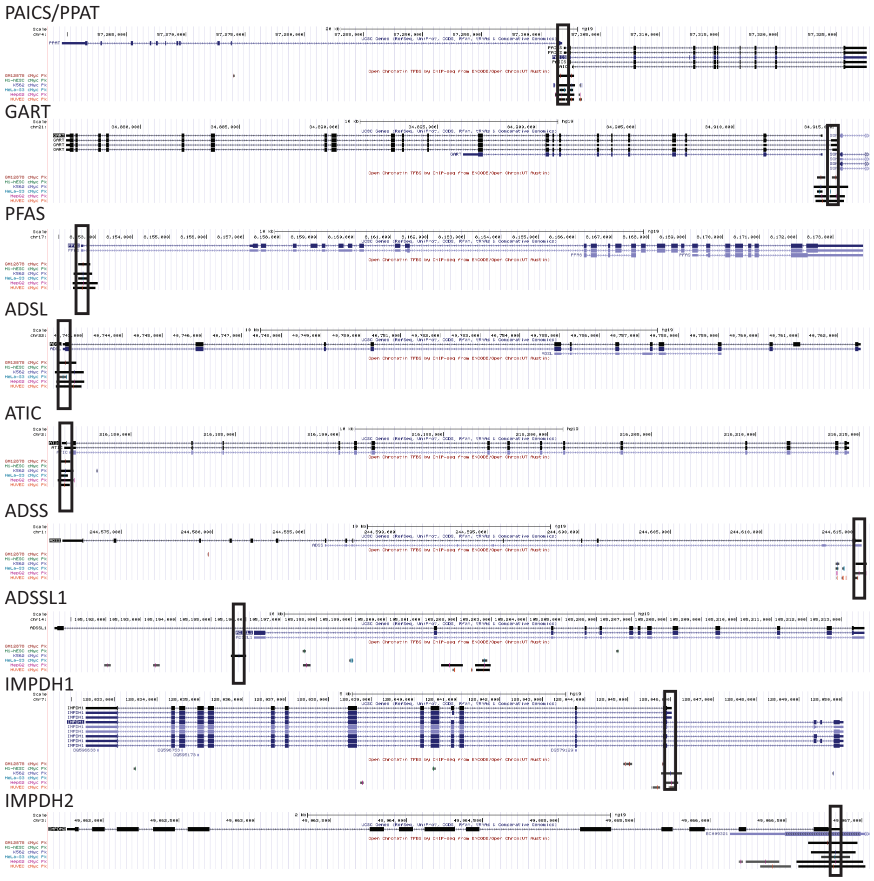

B

real-time PCR primers

	foward	reverse
PPAT	AAATTGTCAGCCCTTCGTTG	CCAGTCTGGGGTGTCATCTT
GART	ACACCGCCCTAACTGTTGTC	GATGGCTGTGACTGCAAGAA
PFAS	TCATGTTTAGTGGGGGCATT	CTGCACAGATGAAGCAGCTC
PAICS	GTCTTCTCTTCGACTACCCAGTGG	CAAATTGAGCTGATCCTTCTGGA
ADSL	TTGCTAAGGAACGAGCCAGT	ATCTCGGACACGCTTCAAGT
ATIC	AACCCTCACACCCATCTCAG	ACATCGGACAATGCAACAAA
ADSS	GGATTTACTGCGTTGGCACT	TGTTCCATCCTGGGAGAGTC
ADSSL1	CGGATCCTTAACACCTGGAG	GCAGCAGGTGGAAGTCGTA
IMPDH1	ATTGGACCTCGCTACACACC	CAGGTAGTCCGCCATGCTA
IMPDH2	AGTGGCTCCATCTGCATTACG	ACCTTGTACACTGCTGTTGCTTG
KLK3	GCAGCATTGAACCAGAGGAG	AGAACTGGGGAGGCTTGAGT
MYC	TACCCTCTCAACGACAGCAG	TCTTGACATTCTCCTCGGTG
DKC1	CGGTGCAGGGTAACATGG	CAATGACTTCCGCTCCTTTT
GEMIN4	CCACTTTCATGGTGTCATGC	GAAGAGCAGCCACTGGAATC
NOP16	GCTAAATCGGTACGGCAGAA	CATAGGGCTTCCGTACAAGC
RRP12	GTACAGCGCTTCTGGGAGTC	TTGTCATCAGAGCAGCGAAG
ACPP	GCCTTGGCTTCTTGTTTCTG	AAGGTGTCAATGGGACTTCG
GPER	AGGGACAAGCTGAGGCTGTA	GCTGAACCTCACATCCGACT
AR	CTCACCAAGCTCCTGGACTC	CAGGCAGAAGACATCTGAAAG
CDKN1A	TGTCTTGTACCCTTGTGCCT	GGCGTTTGGAGTGGTAGAAA

ChIP-qPCR primers

	foward	reverse
PPAT/PAICS	AGCCCTCAGCCCACTTAGGA	GGGTCAGGGAGACCGTGAA
GART	GCCTGCGTCAGACACTAACA	GTCTCTCGCCTTCCTGATTG
PFAS	GTGCAGCGTTTCGTCATCTA	GAATGGACGCCTCCACTAAA
ADSL	CGGCCAAGGAAGTCTTAATG	CTTGGTTCGCTTCTTTGACC
ATIC	TTTTCAGCATGGGAGCTTCT	GGGCAAATGCCTGTTATTCT
ADSS	GTGTATTCTGGGGCCACTGT	CCCTGTCCCCATCTCTGTTA
ADSSL1	CCGCGTGTTTATTTCAGGAT	GGGACTTCAGTCCAGACGAG
IMPDH1	GCCGCTGATCAGGTAGTCC	GTCAGCAGTAGCAGCAGCAG
IMPDH2	TTGAACTGGCCGAGGTCCT	GCACATAGTGGCCTTGTCACTG
negative	AACTCCACATTTCCTAAGTGACC	CCAACCCACACCAAGTACC

Supplementary Figure S2: A. UCSC genome browser tracks depicting the TSS of the examined purine biosynthesis genes. Approximate primer locations are encircled, for details see supplementary methods. **B.** Primer sequences used for qRT-PCR (left) and ChIP qPCR (right).

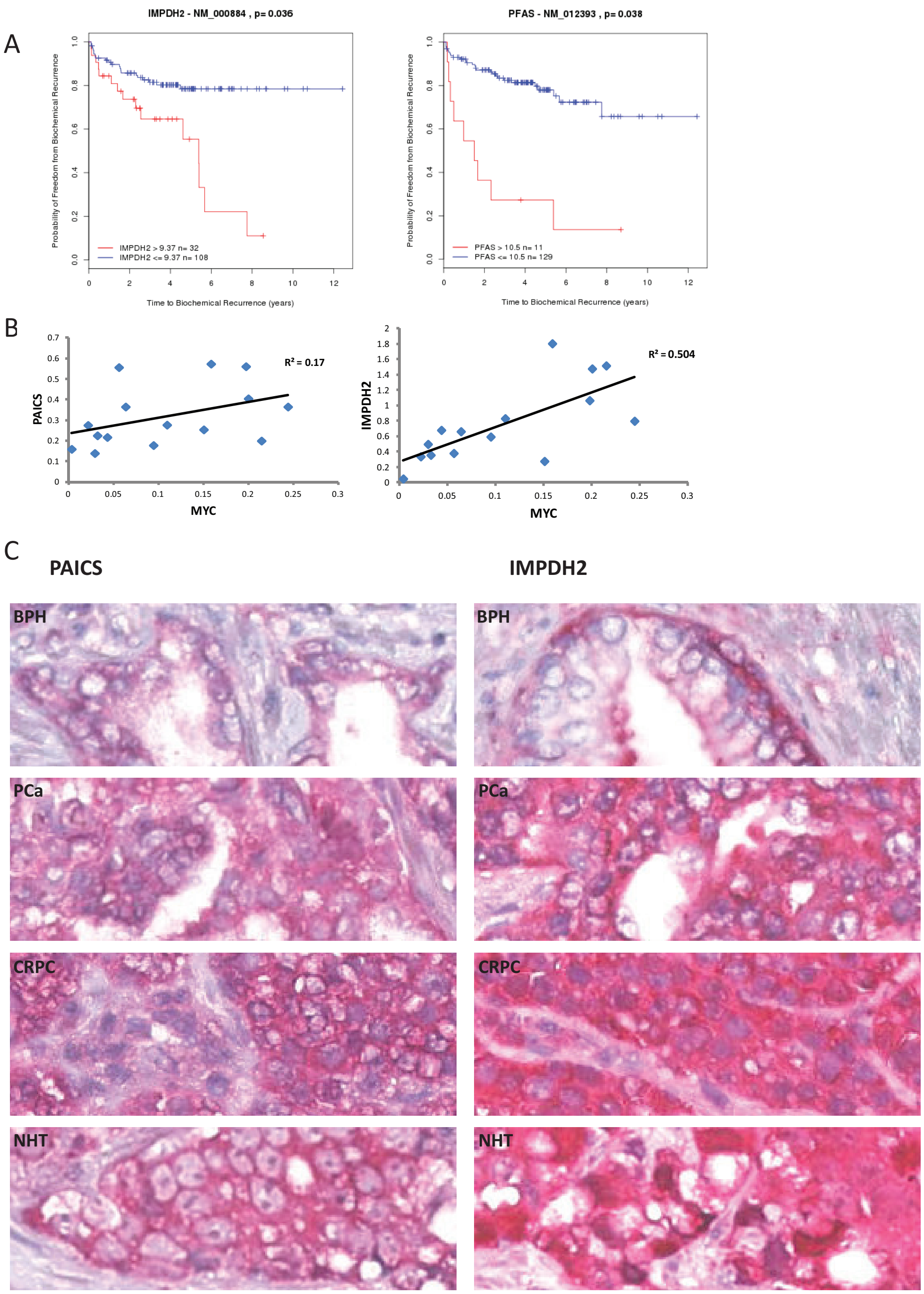

Supplementary Figure S3: A. Significant results of recursive partitioning using the dataset published by Taylor *et al.* performed on all genes involved in purine *de novo* biosynthesis is displayed as Kaplan-Meier survival curves. **B.** Correlation coefficient curve. The log2 normalized expression values of the indicated gene combinations were plotted and a correlation coefficient was calculated to assess coexpression of the respective genes (based on the patient cohort used in Figure 2B). **C.** Representative high-magnification images of the TMAs for PAICS (left) and IMPDH2 (right) shown in Figure 2C/D.

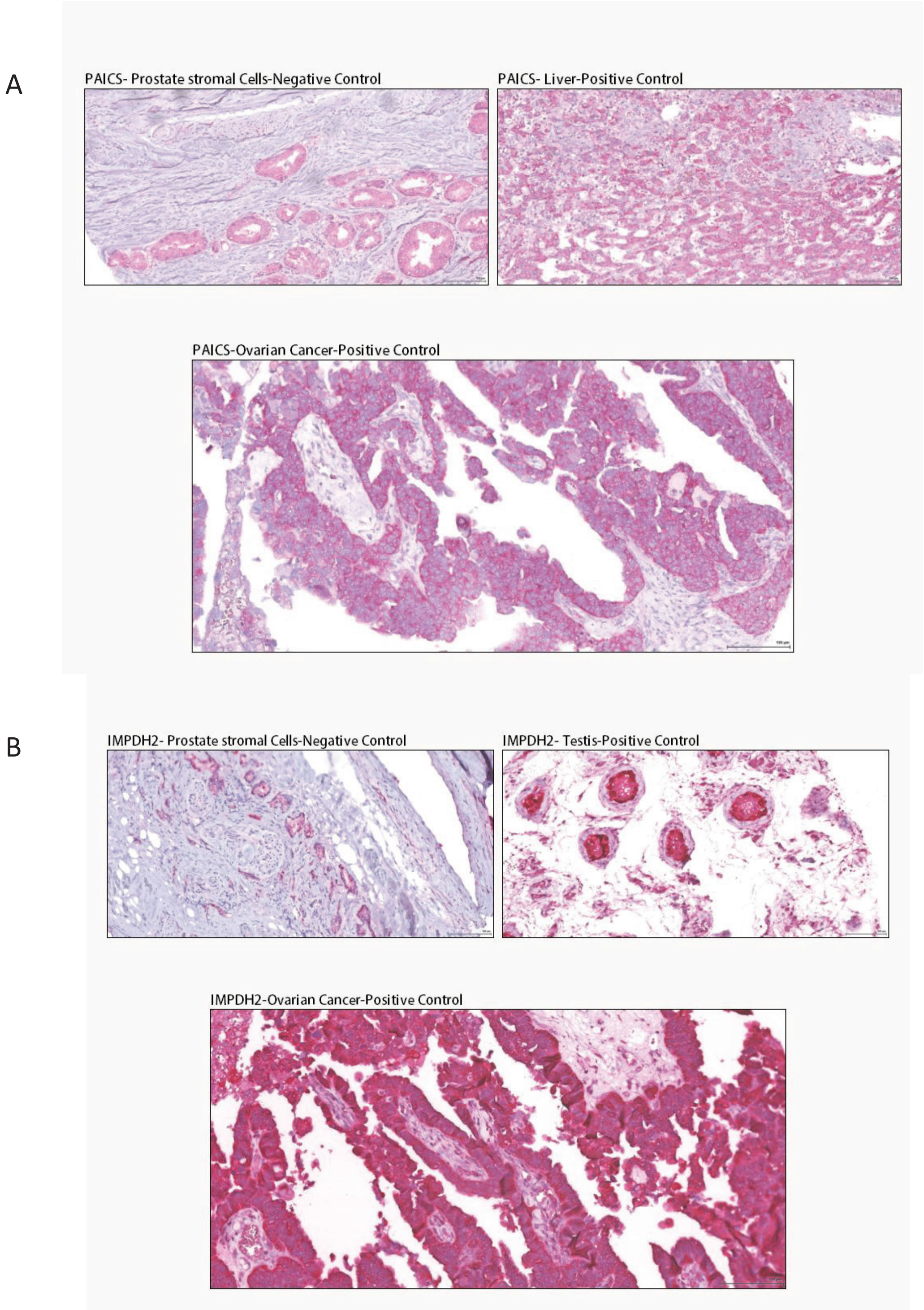

Supplementary Figure S4: Representative images of positive and negative controls for immunostaining of A. PAICS (negative control–prostate stroma and positive controls–liver tissue and ovarian cancer) and B. IMPDH2 (negative control–prostate stroma and positive controls–testis and ovarian cancer).

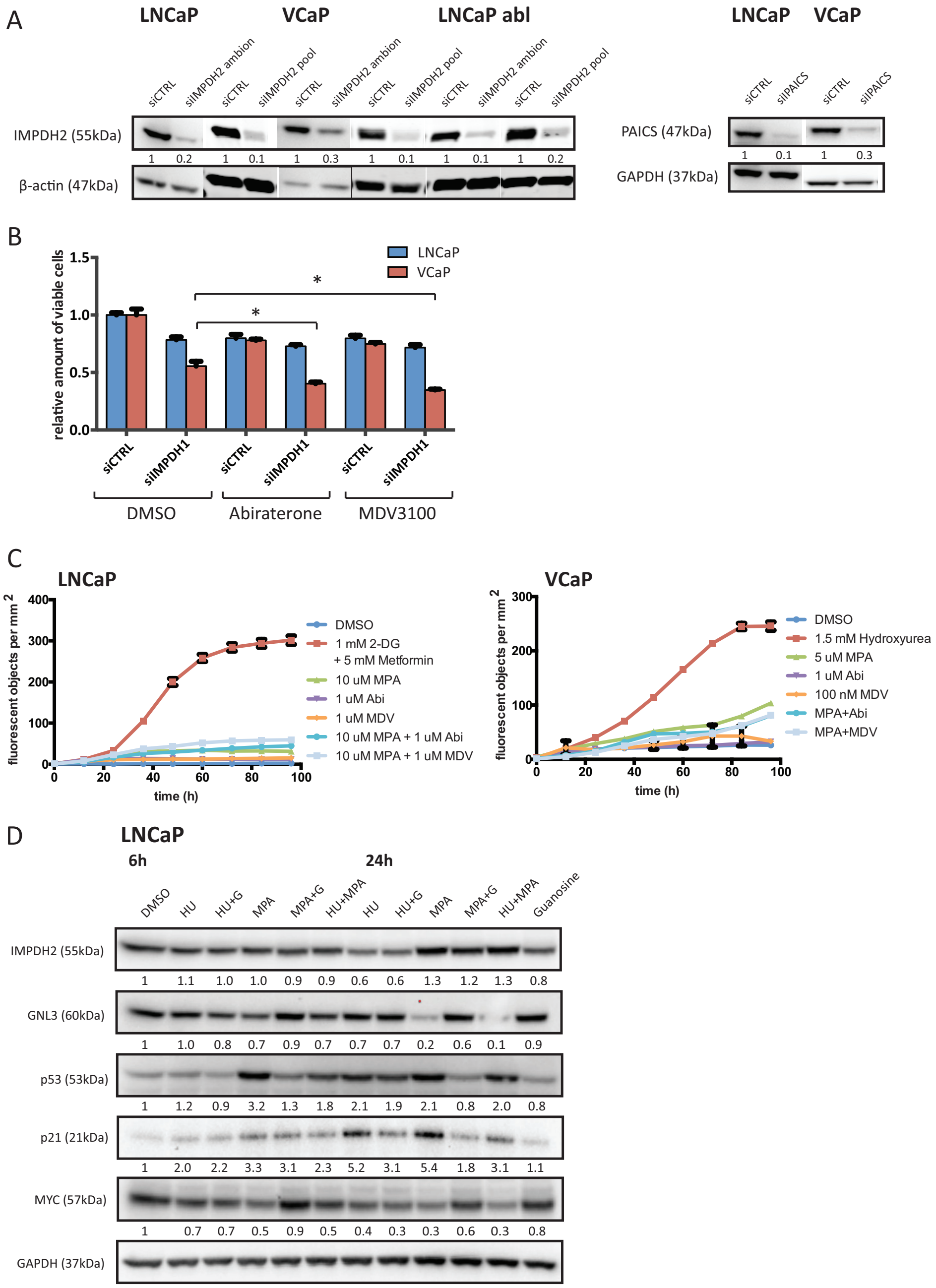

Supplementary Figure S5: A. Knockdown efficacy of siRNA-transfected LNCaP and VCaP cells. LNCaP and VCaP cells were transfected with 25 nM (IMPDH2 ambion) or 50 nM (IMPDH2 pool/PAICS) or equivalent amounts of non-targeting control siRNA (siCTRL) for 72 h. Protein lysates were harvested, separated by SDS-PAGE and blotted for the indicated proteins. **B.** Cell viability results of siRNA and Abiraterone/MDV3100 treated cells. Cells were transfected with 25 nM IMPDH1 or equal amounts of siCTRL for 48 h. Following treatment with the indicated drugs for another 72 h, viability relative to DMSO and siCTRL was assessed using a MTS-based assay. Doses for LNCaP were 1 µM Abiraterone and 1 µM MDV3100, and for VCaP 1 µM Abiraterone and 100nM MDV3100. $n = 3–4$ **C.** Caspase cleavage assay using the fluorescent-based CellPlayer system. LNCaP (top) and VCaP (bottom) cells were allowed to attach for 48 h prior to treatment with the indicated drug combinations. Activation of caspase as determined by fluorescence was monitored for a total of 96 h using an automated imaging system (Incucyte). 2-Deoxyglucose and Metformin (LNCaP) or Hydroxyurea (VCaP) have been shown to induce apoptosis in the respective cell lines and were used as positive controls. **D.** Western Blot analysis of LNCaP treated with mycophenolic acid (10 µM), hydroxyurea (1 mM) or guanosine (100 µM) for the indicated timepoints. Protein lysates were harvested, separated by SDS-PAGE and blotted for the indicated proteins. Protein levels were normalized to 6 h DMSO control and GAPDH.

Supplementary Table S1. The 266 genes upregulated significantly (ranked product analysis, *q*-value < 0.05) by both 5 h and 12 h stimulation with 2 µg/ml Doxycycline in LNCaP MYC cells are shown.

Supplementary Table S2. The 264 genes that correlated with MYC using a Pearson correlation coefficient with 1% tolerance in the Taylor *et al.* dataset are shown. Only patients with BCR and/or metastases were included in the analysis.

Supplementary Table S3. LNCaP MYC were treated with 2 µg/ml Doxycycline for 12 h and the top 10 up- and downregulated gene sets, out of a total of 4772 in the C2: curated gene sets list from the broadinstitute (http://www.broadinstitute.org/gsea/msigdb/collections.jsp#C2), are shown.
